# Evidence of poor adherence to secondary prevention after acute coronary syndromes: possible remedies through the application of new technologies

**DOI:** 10.1136/openhrt-2014-000166

**Published:** 2015-01-24

**Authors:** Kevin Cheng, Nicola Ingram, Jan Keenan, Robin P Choudhury

**Affiliations:** 1 Medical Sciences Division, University of Oxford, Oxford, UK; 2 Department of Cardiac Medicine, Cardiac Investigations Annexe, John Radcliffe Hospital, Oxford, UK; 3 Radcliffe Department of Medicine, Acute Vascular Imaging Centre, University of Oxford, John Radcliffe Hospital, Oxford, UK

**Keywords:** QUALITY OF CARE AND OUTCOMES

## Abstract

Adherence to secondary prevention medications following acute coronary syndrome (ACS) is disappointingly low, standing around 40–75% by various estimates. This is an inefficient use of the resources devoted to their development and implementation, and also puts patients at higher risk of poor outcomes post-ACS. Numerous factors contribute to low adherence including poor motivation, forgetfulness, lack of education about medications, complicated polypharmacy of ACS regimens, (fear of) adverse side effects and limited practical support. Using technology to improve adherence in ACS is an emerging strategy and has the potential to address many of the above factors—computer-based education and mobile phone reminders are among the interventions trialled and appear to improve adherence in patients with ACS. As we move into an increasingly technological future, there is potential to use devices such as smartphones and tablets to encourage patient responsibility for medications. These handheld technologies have great scope for allowing patients to view online medical records, education modules and reminder systems, and although research specific to ACS is limited, they have shown initial promise in terms of uptake and improved adherence among similar patient populations. Given the overwhelming enthusiasm for handheld technologies, it would seem timely to further investigate their role in improving ACS medication adherence.

**Key messages**

**What is already known about this subject?**

- Various studies have shown adherence to secondary prevention medications to be poor after acute coronary syndrome. Data suggest adherence is improved via the use of a limited range of technologies—however, the utilisation of recent technologies (eg, smartphones and tablet apps) has great potential but is not well researched.

**What does this study add?**

- This literature review clarifies the problem facing clinicians in terms of adherence and synthesises what is known about the use of technology in improving adherence after acute coronary syndrome. This review also considers potential future uses of recent technological innovations, drawing attention to the benefits they may offer.

**How might this impact on clinical practice?**

- This may have effects on clinical practice such as to encourage further research into developing ways of promoting adherence through these popular technologies.

## Introduction

Pharmacological interventions, so-called secondary prevention, reduce morbidity and mortality after acute coronary syndrome (ACS).^1^ Clinical trials demonstrate efficacy, and the considered implementation of their findings, aided by guidelines (eg, the National Institute of Health and Care Excellence (NICE)), promote best prescribing practice.^2^ However, to extract benefit, patients must actually take prescribed agents. Defined as ‘the extent to which a person's behaviour—taking medication, following a diet and/or executing lifestyle changes, corresponds with agreed recommendations from a health care provider’, adherence is crucial for the delivery of effective medical treatment.^3^

Current secondary prevention medications include ACE inhibitors (ACEI) or angiotensin receptor blockers, statins, β-blockers and antiplatelet agents. Significant non-adherence has prevented their translation into maximal clinical benefit. Associated with excess mortality and a significant financial burden, the need to address non-adherence to secondary prevention medication after ACS is of the utmost clinical and public health importance.^4^ ^5^

Advances in technology may offer solutions. If they are able to address the multifactorial nature of non-adherence, innovative technological interventions have the potential to engage with patients via interactive interfaces and provide real-time feedback. Additionally, they may be tailored to patient-specific needs. In this review, we will outline the extent and reasons, modifiable as well as non-modifiable, for patient non-adherence, and critically evaluate technological interventions that have been developed to improve secondary prevention after ACS.

## Extent of non-adherence

The WHO estimates that, in developed countries, adherence to medication for chronic diseases averages only 50%.^3^ In particular, adherence to secondary prevention medication after ACS is suboptimal worldwide.^4^ Most studies have investigated secondary adherence (continuation of medication) although some have assessed primary adherence (prescription initiation). In a cohort study in Ontario, Canada, only 73% of patients filled their discharge prescription at 1 week after discharge post-ACS, with increased 1-year mortality associated with fewer initial prescriptions filled.^6^

Short and long-term secondary adherence is also poor.^7–12^ In a multicentre study involving 19 US hospitals (n=2498), 1 month after discharge on aspirin, β-blockers and statins, 34% of patients had stopped at least one and 12% had stopped all three medications.^7^ At 1–2 years, studies have shown only 40.1–45% of patients are adherent to β-blockers or statins with further reductions seen over a 10-year follow-up.^8–10^

Importantly, mortality increases with non-adherence. Increased mortality is associated with poor β-blocker or clopidogrel adherence at 1 year (2.6-fold increased mortality or non-fatal myocardial infarction), and statins and β-blockers in the longer-term (median 2.4 years of follow-up).^5^ ^12–14^ In addition, over median follow-up of 23 months, a prospective cohort study using the Finnish Prescription Register demonstrated a dose–response relationship between (1) regular (2) irregular or (3) no statin use and mortality (4.9%, 9.4% and 14.9%, respectively), and cardiovascular death in particular (2.9%, 5.1% and 7.4%).^14^ Non-adherence to dual antiplatelet therapy (aspirin and clopidogrel) after stent implantation also poses the added risk of potentially catastrophic stent thrombosis.^15^

It is worth noting, however, that these figures merely give an indication of adherence, which varies between study population according to demographics and means of measurement (eg, patient self-reporting vs assessing prescription refills).^16^ Healthier behaviour in general (‘healthy adherer effect’) may also be a contributing factor—indeed, non-adherence to placebo is associated with a twofold increase in mortality compared to placebo adherence.^17^ Some estimates are more conservative—Eagle *et al*^18^ reported lower discontinuation rates at 6 months (8% for aspirin, 12% β-blockers, 20% ACEI and 13% for statins); perhaps a reflection of a younger study population. Even by these measures, however, the scale is considerable and warrants improvement.

## Reasons for non-adherence

Although diverse and often coexisting reasons make it difficult to identify why ACS secondary prevention regimens are not adhered to, certain factors have been consistently associated with poor medication adherence. Patient perception of the medication regimen tallies with adherence, with those patients rating (1) high on a scale of perceived necessity of medicines prescribed for ACS and (2) low on a scale of concerns about side effects being less likely to miss doses.^19^ Similarly, patient-reported reasons for discontinuation of ACS medication include the belief that it was not helping with their condition.^20^ Patient motivation also appears a significant issue, and depression (though not anxiety) persistently correlates with lower adherence to ACS medications.^21^ ^22^ Severity of depression was even associated with the extent of non-adherence in one particular study.^21^ Furthermore, a change from the patient's baseline depressive symptoms brought about an improvement in adherence.^21^ ^22^ Considering an estimated one-third of patients with ACS suffer from depression, this would appear an important area to address.^21^

A motivational issue reported by patients is simply forgetting to take their medications: in a study of 190 patients with ACS, 23.2% declared this as a reason on the Morisky Medication Adherence Scale (MMAS) questionnaire (an 8-item self-reported adherence questionnaire).^23^ This may in part be a reason for the poor adherence associated with the prescription of multiple medications.^23^ ^24^ Up to six medications may form the post-ACS regimen alone and lack of reminders (such as pillboxes and calendars) was also significantly associated with poor adherence.^24^ Similarly, patients with fewer practical supports (in particular a spouse) are less likely to be adherent, possibly due to a lack of encouragement or prompting.^25–27^ The level of expertise that patients are exposed to in the rehabilitation setting (eg, cardiologist or tertiary care) may also play a part: Eagle *et al*^18^ noted that while adherence to aspirin was 92% at 6 months, analysis showed this to be associated with care by a cardiologist rather than a non-specialist.^6^ ^28^ A similar association has been demonstrated in patients with heart failure, where improved survival was seen in patients under cardiologist or mixed general practitioner (GP) and cardiologist care compared to GP care alone.^29^ Since these studies rely on simple correlation between variables and may be biased by using patient self-reporting, these results must be treated with caution, but the effects of these factors, replicated across studies, are sufficiently great to be recognised by the patients themselves. Furthermore, the above factors are modifiable: better education for patients and their carers about medications, treatment for depression and a system of reminders may all contribute to improved adherence.

Additional non-modifiable factors affect adherence. Perhaps the most serious deterrents are the adverse effects of the medications themselves, as evidenced by the poorer adherence among patients on clopidogrel who experienced episodes of bleeding (HR 1.34 for discontinuation of clopidogrel after experiencing bleeding).^12^ The presence of comorbidities also tallies with the likelihood of taking medications post-ACS—the low adherence group studied having an average of 3.8 coexisting conditions compared with 1.3 in the high-adherence group.^23^ Older age has been consistently linked with lower adherence to medications prescribed after an episode of ACS; self-reported reasons for this include side effects being a greater deterrent in the older population.^12^ ^20^ ^23^ Gender does not appear to correlate with poor adherence but there is a suggestion that patients with higher educational status or who are employed are less likely to discontinue medications.^23^ ^24^ While they may not be directly influenced, these factors may be considered when tackling adherence, such as better education about side effects, and may be tailored to what studies show is a broadly similar demographic between populations of patients with ACS (mean age around 60 with a male preponderance). There are, however, international differences depending on healthcare system: cost of medicines may, therefore, be a major deterrent to adherence in the US studies but not such a problem encountered, for instance, in the UK National Health Service (NHS) system, which should be taken into consideration when interpreting these studies as well as when designing programmes to improve adherence.^20^ ^24^

## Technological interventions in ACS

In addressing these modifiable factors, innovative technological interventions may hold the key.^30–33^ However, increasing the number of components poses many difficulties not least logistically but financially. Consequently, technology may offer the potential to meet these requirements, especially with advances that allow greater patient interaction and real-time feedback, features that may also serve as an extension of physician contact outside of the clinic. Many studies to date, however, have only investigated simple interventions consisting of one or two components (table 1). First, we will review interventions that have been trialled to improve adherence to secondary prevention medications after ACS and then consider future innovative technological directions.

**Table 1:** Studies investigating the effect of technological interventions on adherence to secondary prevention medications after acute coronary syndromes

Study	Technological intervention	Study population	Medications	Design	Study size (mean age)	Duration	Main adherence measure	Main findings
Quilici *et al*^34^	Mobile-phone—daily personalised SMS reminders	Patients who had undergone coronary stenting for ACS with good in-hospital aspirin response defined by AA-Ag lower than 30%	Aspirin	RCT	546 patients (intervention: 64±10; control 64±14)	1 month	Self-reporting and AA-Ag testing	Improved medication adherence with daily SMS reminders—self-reported aspirin non-adherence: 3.6% (SMS intervention) vs 6.4% (standard care) p=0.02; AA-Ag testing: 5.2% SMS intervention) vs 11.2% (standard care); p=0.01
Rinfret *et al*^38^	Telephone contact by nurses—4 sessions (<7 days, 1, 6 and 9 months) after stent implantation	Patients who had undergone drug-eluting stent implantation	Aspirin and clopidogrel	RCT	300 patients (64±10)	12 months	Proportion of days covered with aspirin and clopidogrel as assessed by pharmacy refill data	Significant improvement in adherence with four telephone calls—median scores for aspirin (99.2% vs 90.2%; intervention vs standard care respectively) and clopidogrel (99.3% vs 91.5%); p<0.0001 for aspirin and clopidogrel
Ho *et al*^39^	Multifaceted—including voice messaging (educational and medication refill reminder calls)	Patients admitted with ACS	Clopidogrel, β-blockers, statins, ACEI/ARB	RCT	253 patients (intervention: 63.8±9.25; control: 64.0±8.57)	12 months	Proportion of patients adherent to medication regimens based on mean proportion of days covered greater than 0.80 using pharmacy refill data	Increased adherence—89.3% vs 73.9% of patients were adherent (intervention vs usual care); p=0.003

AA-Ag: arachidonic acid induced platelet aggregation; ACEI: angiotensin-converting-enzyme inhibitor; ACS: acute coronary syndromes; ARB: angiotensin receptor blocker; RCT: randomised controlled trial; SMS: short messaging service.

### Electronic reminders

Such interventions have focused on daily text messaging (short message service—SMS) and follow-up telephone calls. Evidence suggests that patients respond to prompting—Quilici *et al*^34^ investigated the use of personalised daily motivational SMS messages in patients after coronary angioplasty. Using SMS messaging to remind patients to take their antiplatelet medications, self-reported non-adherence significantly improved over 1 month (OR 0.37). Importantly, to mitigate recall bias, platelet function testing (via arachidonic acid-induced platelet aggregation) was used to provide objective, biological data, which confirmed these improvements.^34^ While SMS messaging may be cheap and allows scope for tailored intervention, patient education may not necessarily be improved—patient education sessions were still provided on discharge to emphasise the importance of adherence. Paternalistic in nature, they do not encourage patients’ own responsibility and initiative to improve their own health, something more interactive interventions may offer.

Limitations to this study also include its short time period (1 month), lack of a clinical endpoint as well as the use of complete aspirin discontinuation as non-adherence—more subtle changes, for example, missed doses, were not accounted for. Intriguingly, compared to previous studies, the proportion of patients on usual standard care who had stopped aspirin was low—6.4% (self-reported) and 11.2% (platelet function test)—a feature commonly seen in randomised controlled trials compared to cohort studies. The results of a longer-term follow-up would be of particular interest in light of a systematic review by Vervloet *et al*^35^ that also reported short-term (<6 months) but not long-term effectiveness of electronic reminders.

### Technology-assisted patient education

These range from the basic, for example, telephone calls and voice messages, to more complex but unevidenced interventions such as smartphone or tablet (eg, iPad) applications (apps). Such ‘telehealth’ interventions have shown promise.^36^ ^37^ Indeed, nurse-led telephone calls, in a recent randomised controlled trial, significantly improved 1-year adherence to dual antiplatelet therapy after drug-eluting stent implantation for ACS.^38^ Four phone calls (within 7 days, 1, 6 and 9 months after stent implantation) assessed adherence to dual antiplatelet therapy and emphasised its importance, including the risks of non-adherence. Measured as the proportion of days covered by prescription refills, phone calls significantly improved median adherence scores—aspirin 99.2% vs 90.2%; clopidogrel 99.3% vs 91.5% (p<0.0001 for both). Indeed, 87.2% of patients were persistent on clopidogrel at 1 year compared to 43.1% of controls. Limitations, however, include its setting in a tertiary care university cardiovascular centre and a surrogate score for adherence (pharmacy refills). There were no clinical end points and adherence scores were high relative to other studies.

Where resources allow, a combination of the above strategies may prove most beneficial. A recent study has shown improved adherence in a group of patients with ACS receiving a package of education, voice messages (educational and reminder) and a pharmacist-led tailored medication regimen compared with the usual care group (89.3% vs 73.9%).^39^ There is some doubt as to whether this modest improvement justifies the cost of implementing such a comprehensive programme, and the case would be strengthened if these results were to be replicated and clinical benefit demonstrated.

Furthermore, a plethora of apps for smartphones and tablets is available on the market. Not only can these apps provide information on cardiac health but they can also act as diaries and reminder systems. Although potentially useful as patient aids, evidence of clinical benefit from them is largely anecdotal.

## Future directions

Considering the current shortage of studies particular to ACS, it is necessary to look to technologies aimed at similar population groups, for instance heart failure cohorts. These range from simple electronic prompts to more complex, interactive packages of education, health records and reminders accessible via smartphone and tablet devices (table 2). Didactic approaches, such as an alarm system to alert patients when medication is due, may have some effect. An audiovisual reminder attached to the blister packs of medications of a group of patients with hypertension produced better adherence to the antihypertensives (91% vs 85%) but had no effect on blood pressure values.^40^ Furthermore, around 50% of the patients approached chose not to use the device (and the high adherence values of the consenting group suggested this was an already motivated cohort), hence this may not be the optimal way of engaging patients most in need of help with medication adherence. Technology-assisted education may better involve patients: heart failure patients who completed a course of computer-based education modules and quizzes were subsequently more informed about their condition than controls.^41^ There was no difference in either quality of life or medication adherence scores, however, suggesting that education alone is not sufficient.

**Table 2:** Advantages and disadvantages of technologies designed to improve chronic medication adherence in cardiovascular disease

Technology	Advantages	Disadvantages
Medication container alarms^39^	Simple, cheap, evidence for improved adherence	Poor uptake
SMS reminders^35^ ^36^	Cheap, convenient for patients, evidence for short term benefit on adherence	No proven effect in long term
Nurse-led telephone calls^37^	Patient-specific, evidence for improved adherence	Resource-heavy in terms of staff hours
Computer-based education modules^40^	Encourages patient participation, patients better informed about their condition	May be inconvenient for patients, no evidence of improved adherence.
Interactive Voice Response technology^41^	Useful for large numbers of patients, can create tailored responses, evidence for improved adherence	May be viewed as impersonal
Online records^42^	Convenient for patients	Risk of confidentiality breach, no evidence of improved medication adherence
Smartphone/tablet applications^43–45^	Interactive, tailored to individual patients, evidence for improved adherence	Expensive, may not be accessible to all cohorts of patients

More recently, there has been a move to personalised education according to a patient's prior knowledge and individual needs. Interactive Voice Response (IVR) technology is particularly useful in providing this broad assessment and has shown promise among long-term medication users. A study of statin users demonstrated that patients who received guidance based on their responses to questions regarding knowledge, beliefs about their condition and reasons for non-adherence, were more adherent to medications at 6 months compared to those who received generic information (70.4% vs 60.7%).^42^

Integration of the internet into our daily lives has done much for patient empowerment in terms of readily accessible information. Additionally, there is scope for making personal medical records available to patients at home, and programmes such as SPPARO (System Providing Access to Records Online) have shown that this improves adherence to general medical advice among a group of patients with heart failure, if not to the medications themselves.^43^

It is the rise of apps and handheld devices such as smartphones and tablets, however, which is likely to inform development of aids to adherence in the near future. Ease of use, portability, storage and versatility of function, along with the fact that they are often already a part of the patient's life, make these technologies ideal in the rehabilitation setting. Commercially available apps have shown promising uptake: a German-language ‘Medication Plan’ app was reportedly used by 11 688 users, 74% of whom had cardiovascular disease.^44^ ^45^ Indeed, medical organisations have been quick to recognise this and have called for expansion in this area: one US competition—the Aetna CarePass Developer Challenge—challenges designers to produce a mobile phone application specifically assisting patients with adherence to medication regimens.^46^

Much of medication app research, unfortunately, concentrates on different populations (eg, patients with diabetes mellitus), hence there may be limited basis for predicting how patients with ACS would respond to this technology.^47^ However, similarities include population age and the chronicity of these diseases, which indeed may coexist. Consequently, consideration of these studies such as features affecting their usability, for example, multifunctionality, documentation or analysis functions (which all reduce usability), may aid the development of apps for patients specifically post-ACS.^47^ ^48^ A potential disadvantage, such as seen with the low take-up of electronic reminder devices, is the perception of this technology as an unnecessary complication: in an evaluation of various mobile medication management applications by older adults, study participants reported on the whole that they did not feel a need for these applications in their own medication regimes.^40^ ^48^ Usability was not a problem, however, possibly due to the designers’ use of clear wording and simple navigation (modifications such as large font sizes may also be of benefit, and can be dictated by individual requirements).

Other studies have produced more encouraging results: a tablet-based pillbox app helping to remind elderly patients with chronic illness to take medications, along with providing lifestyle advice, resulted in improved MMAS scores, and the concept was met with approval by the patients themselves (28.3% rise in adherence; mean satisfaction score 8.5 of 10).^49^ Similarly, a pilot study gauging interest in a tablet programme providing education on heart failure medication adherence in a text, video and quiz format was generally welcomed by patients and nurses. Patients also reported a need for empathetic rehabilitation, interactive learning, and support from healthcare workers and family members, of which app technologies may be one unifying aid.^50^ The challenge is, therefore, to design apps that are easy to use and informative, yet are not viewed as an encumbrance by the patients who could benefit from them.

## Conclusions

Given the scale of non-adherence to ACS secondary prevention and the mounting wealth of technologies at our disposal, there would appear to be scope for designing cost-effective programmes to improve medication adherence. Current estimations of adherence in ACS stand at around 40–75%, though this figure is dependent on follow-up time and definition of adherence.^6^ ^7^ ^9–12^ Non-adherence, however, translates into poor clinical outcomes.^12–15^ Understanding the reasons for non-adherence, therefore, can inform development of technological methods to improve adherence. Insufficient knowledge about medication regimens, low motivation or forgetfulness, polypharmacy, adverse side effects, increasing age and a lack of practical support have all been implicated and may be borne in mind.^19–24^ In the particular context of ACS, studies have used text or voice message reminders and telephone education to demonstrate better adherence.^34^ ^38^ ^39^

Evidence from other chronic conditions suggests that patients are more likely to engage with technology already at their disposal (the compliance for using separate alarms, for instance, was low) and are more likely to respond to personalised education.^41^ ^42^ The ubiquity of smartphones and tablets may thus provide the ideal rehabilitation opportunity. Such applications have been shown to be popular among patients (including older generations) and to encourage adherence.^49^ ^50^ Research into such technologies is still in the early stages (and especially limited in the context of ACS), but it is likely they will play a major part in the future of cardiac rehabilitation.
